# Construction of an evaluation index system for undergraduate nursing teachers’ curriculum humanistic competence: a Delphi study in China

**DOI:** 10.1186/s12912-023-01432-4

**Published:** 2023-08-25

**Authors:** Zihan Yang, Huimin Zhai, Sijing Liang

**Affiliations:** School of nursing, Southern medical university, NO.1023 Shatai Road, Guangzhou city, Guangdong Province 510515 China

**Keywords:** Education, nursing, Curriculum humanities, Competence, Evaluation indicators

## Abstract

**Background:**

The curriculum humanistic competence of nursing teachers is important to cultivate the humanistic qualities of undergraduate nursing students. However, there are no evaluation tools for the curriculum humanistic competence of undergraduate nursing teachers in China.

**Objective:**

To develop an index system to evaluate the curriculum humanistic competence of undergraduate nursing teachers.

**Design:**

This research conducted a Delphi study.

**Participants:**

Semi-structured interviews were held with 19 experts, and Delphi rounds were conducted with 18 experts.

**Settings:**

This study was conducted in 12 universities and 4 Grade A tertiary hospitals in China.

**Methods:**

A literature review and semi-structured interviews were conducted to develop an initial framework. A two-round Delphi survey was employed to build the index system for undergraduate nursing teachers’ curriculum humanistic competence.

**Results:**

After two rounds of consultation, the index system included 5 first-level indicators, 11 second-level indicators and 41 third-level indicators. The Cr for two rounds of consultation were 0.929 and 0.923, and Kendall’s W was 0.152(*P*<0.001).

**Conclusions:**

The index system for the evaluation of undergraduate nursing teachers’ curriculum humanistic competence offers guidelines for undergraduate nursing teachers in China. It can be used in practice to develop high humanistic qualities in undergraduate nursing teachers.

## Introduction

Caring, which is the basis of nursing, runs through the whole nursing process [1]. Nursing involves both natural and humanistic attributes, so nurses must have not only solid professional skills but also high levels of humanistic qualities [2–4]. Undergraduate nursing students are the backbone of future nursing services, and their humanistic qualities are directly related to the quality of nursing services [5, 6]. Strengthening humanistic qualities in the education of undergraduate nursing students and improving their practical humanistic abilities is not only an important way to adapt to the transformation of today’s medical model and social development but also an important way to boost the humanistic spirit in medicine.

The president of the People’s Republic of China stressed that it is necessary to adhere to moral education as the central link and infiltrate ideological and political work into the whole process of education and teaching. Since then, “curriculum thought and politics” has been put forward. Drawing lessons from the idea of “curriculum thought and politics” and innovating it, a scholar put forward the concept of “curriculum humanities”; that is, humanistic elements are integrated into the classroom teaching and clinical practice teaching of nursing specialties to consistently cultivate humanistic qualities [7]. “Curriculum humanities” provides a new idea for the cultivation of the humanistic qualities of nursing undergraduates, in which humanistic elements are imperceptibly integrated into professional courses to compensate for the lack of a pure humanities curriculum. At the same time, it also puts forward certain requirements for the humanistic qualities of nursing teachers.

However, at present, it is obvious that undergraduate nursing teachers’ humanistic cultivation is not sufficient for the development of nursing education. Undergraduate nursing educators lack formal faculty development to prepare them to teach interprofessional collaboration. Moreover, they have cognitive limitations, and they are incompetent at obtaining and applying information to help them integrate humanistic elements into their curriculum [8]. Studies show that 31% of nursing instructors were not prepared for clinical education, and 26% were not prepared to evaluate students in a clinical setting [9]. Undergraduate nursing teachers are essential to cultivating the humanistic qualities of undergraduate nursing students. For example, they can help students effectively acquire the knowledge, attitudes, and skills necessary for the practice of nursing [10]. Teachers are also important for students’ development of professional values and competence [11–13]. Therefore, it is important to improve the “curriculum humanistic competence” of undergraduate nursing educators and help them adapt to humanistic teaching in the curriculum.

The theory of competence was first proposed by McClellan, which gave birth to the iceberg model of competence [14]. The onion model developed from the iceberg model, but the former pays more attention to the hierarchy [15]. Based on the onion model, using content analysis and semi-structured interview to develop a framework for undergraduate nursing teachers’ curriculum humanistic competence can improve the quality of nursing teaching [16–18]. However, there is currently no relevant research on evaluation tools for curriculum humanistic competence in China.

This study aimed to construct a framework to evaluate undergraduate nursing teachers’ curriculum humanistic competence by using the Delphi method to provide methods of humanistic teaching in the admission, assessment and evaluation stages of education.

## Methods

### Ethical considerations

This study adhered to the tenets of the Declaration of Helsinki. This study was approved by the Ethic Committee of Southern Medical University (approval number: NFYKDX003). Informed consent was provided and obtained from all participants before the study commenced.

### Identification of the advisory experts

The number of Delphi consultants should be controlled at 15–30 to avoid homogeneity of research objects [19]. From September 2022 to December 2022, the research group distributed questionnaires to 20 experts in nursing education, humanistic nursing, nursing teaching management and other fields for expert consultation. The inclusion criteria were the following: (1) Bachelor’s degree or above; (2) Title of deputy high or above; (3) Engaged in undergraduate nursing education, humanistic nursing, nursing teaching management and other related fields for more than 10 years; (4) Have certain research achievements in humanistic nursing, nursing education or nursing teaching management and other related fields and can objectively and comprehensively give suggestions and guidance; (5) Interest in this topic and willingness to participate. The exclusion criteria were the following: (1) Failing to complete the questionnaire according to the content of the questionnaire; (2) Failing to fill in and return the questionnaire within the specified time.

### Index system construction and questionnaire preparation

In the early stage of this study, a systematic literature review was conducted, and content analysis was used to extract elements in the literature related to undergraduate nursing teachers’ curriculum humanistic competence [8, 9, 20–23]. Firstly, as the minimum unit of analysis, sentences were selected based on the following principles: (1) Include a specific concept for undergraduate nursing teachers’ competence; (2) Describe the humanistic competence of undergraduate nursing teachers clearly; (3) Provide specific explanations of the methods of undergraduate nursing teachers possess in curriculum humanistic teaching. A category system was subsequently conducted based on the onion model. The first-level indicators were initially determined according to the division of competences in the onion model. The selected analysis units were coded and summarized using content analysis and were put into the corresponding first-level indicators as the second-level and third-level indicators. At this stage, we formed 5 first-level indicators, 11 second-level indicators and 36 third-level indicators. Then, we conducted semi-structured interviews with 19 experts (5 part-time clinical nursing teachers, 9 full-time college nursing teachers and 5 experts in humanities-related fields) who taught medical undergraduates to enrich the content of the index system. Some of the main questions they were asked were as follows: Have you heard of “curriculum humanities” and how do you understand it? Have you conducted curriculum humanistic teaching? Would you show us specific examples? Would you please share with us any good experience or practices you have had during curriculum humanistic teaching? What are the problems and challenges you have encountered during the process of curriculum humanistic teaching? What support do you need? Do you have suggestions for improving nursing teachers’ curriculum humanistic competence? After the interviews, we converted the recordings to texts within 24 h. The grounded theoretical methods of Strauss and Corbin were used to analyze the texts [24]. Finally, we formed 5 first-level indicators (knowledge base, skills level, professional attitudes, personality traits and intrinsic motivations), 11 second-level indicators and 40 third-level indicators.

The questionnaire consisted of three parts: (1) Guidance to clarify the content of the questionnaire and purpose of the study. (2) Inviting experts to evaluate the text content of the indicators of the questionnaire. In this part, a 5-level Likert scale was used to evaluate the indicators. Five points means very important, 4 points means important, 3 points means neutral, 2 points means unimportant, and 1 point means very unimportant. Experts could put forward their views in the corresponding position of the questionnaire. (3) Obtaining information from experts, including their ages, education background, professional title, research field, working years, judgment basis for indicator importance (Ca) and familiarity with indicator content (Cs).

### Process of consultation

The research group distributed the questionnaire to the experts in the form of e-mail and reminded them to reply within 10 days. After the first round of consultation, the research group analyzed the questionnaires. In combination with screening criteria, expert opinions and group discussion results, the indicator system was supplemented, deleted and modified to form the second round of the expert consultation questionnaire. According to the score of each indicator, we calculated the mean value, full score rate and coefficient of variation in its importance assignment. The elements could be included when the coefficient of variation was < 0.25 and the mean value of importance assignment was > 4.00 [5]. If the above requirements were not met at the same time, the final selection of structural elements was determined based on the expert’s modification suggestions and the discussion of the research group. When the experts’ approval rate of the curriculum humanistic competence evaluation framework of undergraduate nursing educators was > 70%, the consultation was over [25].

### Statistical methods

SPSS26.0 was used for descriptive statistical analysis of the data. The continuous data were expressed by means ± standard deviations, and the categorical data were expressed by frequencies and percentages [26]. The reliability of the experts was tested by the authoritative coefficient (Cr) [27] and the expert coordination coefficient (Kendall’s W) [28]. The experts’ evaluation of items was expressed by the coefficient of variation (CV), mean of importance assignment, and full score rate (CLV) [28]. The difference was statistically significant at *P* < 0.05.

We used the analytic hierarchy process to calculate the importance of each item of the 2round Delphi consultation. YAHP12.9 software (Shanxi Yuan Decision Software Technology Co., Ltd., Shanxi, China) was used to establish a judgment matrix and calculate the weights of the indicators.

## Results

### Information from the experts

The experts for the first round of Delphi consultation were from 12 undergraduate colleges and 4 Grade A tertiary hospitals in 10 provinces and municipalities directly under the Central Government. In round 2, we distributed questionnaires to 18 experts who responded in the first round, and 16 responded (the response rate of experts reached 88.89%); they were from 10 undergraduate colleges and 4 Grade A tertiary hospitals in 9 different provinces and municipalities directly under the Central Government. The characteristics of the experts are shown in Table 1.

**Table 1:** Characteristics of the participants

Characteristics	Round 1 (n = 18)			Round 2 (n = 16)	
	n or (Mean ± SD)	Percentage		n or (Mean ± SD)	Percentage
Age(years)	49.89 ± 5.93			49.75 ± 5.41	
Education background					
Doctor	2	11.11		1	6.25
Master	14	77.78		13	81.25
Bachelor	2	11.11		2	12.50
Professional title					
Senior	7	38.89		7	43.75
Deputy senior	10	55.56		8	50.00
Intermediate	1	5.556		1	6.25
Research field					
Nursing education	11	61.11		10	62.50
Nursing management	3	16.67		3	18.75
Medical humanities related fields	4	22.22		3	18.75
Working years	27.44 ± 7.44			27.50 ± 6.86	

### Reliability of the expert panel

Authoritative coefficient (Cr) = (Ca + Cs)/2. In this study, the Cr of round 1 is 0.929, and the Cr of round 2 is 0.923. The scores showed that the results were highly credible [27]. For the two rounds of consultation, the scores of Kendall’s W are shown in Table 2, which suggested that the expert opinions had great consistency.

**Table 2:** Coordination results of expert opinions

	Indicators(n)	Kendall’s W	*χ²*	*P*
First round				
Total	56	0.152	150.031	<0.001
first-level indicators	5	0.259	18.667	0.001
second-level indicators	11	0.118	21.201	0.020
third-level indicators	40	0.154	108.019	<0.001
Second round				
Total	57	0.152	152.821	<0.001
first-level indicators	5	0.313	22.519	<0.001
second-level indicators	11	0.107	19.257	0.037
third-level indicators	41	0.153	110.231	<0.001

### Results of expert consultation

#### Delphi Round 1

In round 1, all indicators met the requirements (mean, 4.50 ~ 5.00; SD, 0.00 ~ 0.59; CV, 0.00 ~ 013; CLV, 50%~100%). In this round of expert consultation, experts stated that narrative nursing was one of the nursing methods, and the “narrative nursing ability” was not equal to the previous humanistic abilities. Therefore, we deleted the third-level indicator “narrative nursing ability”. In addition, experts pointed out that the sense of achievement and gain is important in the motivation of undergraduate nursing educators’ curriculum humanistic competence. Therefore, the “sense of competence” entry was added under the second-level indicator “achievement motivation”, and the third-level indicator “others’ respect” was changed to “others’ respect and recognition”. After modification, round 2 was started.

#### Delphi Round 2

**Fig. 1:**
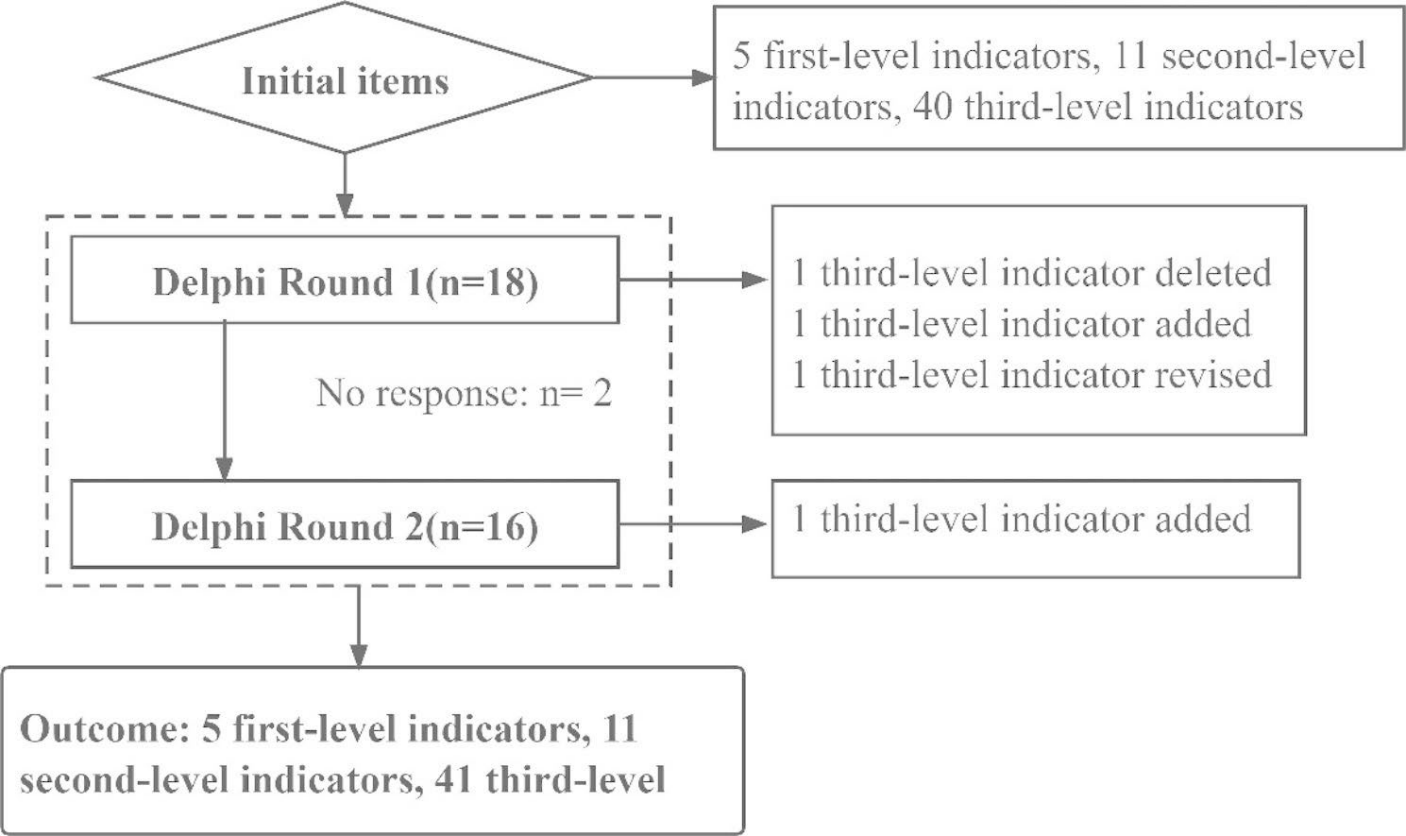
Consultation process flowchart

In round 2, each index value was within the boundary value range (mean, 4.44 ~ 5.00; SD, 0.00 ~ 0.61; CV, 0.00 ~ 013; CLV, 44%~100%). Experts proposed two changes. They suggested supplementing the third-level indicator “reverence for life” to expand the connotation of the second-level indicator “professional emotion”. Therefore, we added one three-level indicator. At the same time, they stated that the second-level “clinical practice ability” has little to do with curriculum humanities and suggested deleting it. Clinical practice teaching is an indispensable link in undergraduate nursing teaching. After comprehensive consideration, we retained “clinical practice ability” and 5 third-level indicators under it. Then, 5 first-level indicators, 11 second-level indicators and 41 third-level indicators were finally established (Fig. 1).

#### Weight analysis

Using the analytic hierarchy process, the weight of each indicator was determined. We built a hierarchical structure model and established a judgment matrix to calculate the weight. In the hierarchical structure model, the target level is the index system for humanistic competency evaluation for undergraduate nursing teachers. The criterion level is composed of 5 first-level indicators and 11 second-level indicators. The scheme level is composed of 41 third-level indicators. After that, we built a judgment matrix. When CR < 0.1, the judgment matrix is satisfactory [5]. After the judgment matrix met the requirements, we calculated the weights of the indicators (Table 3).

**Table 3:** Weight of the indicators

Level of indicators	Name of indicators	Weight	Portfolio weight	CR
I	Knowledge base	0.3077		<0.001
I-1	Professional nursing knowledge	0.6667	0.2052	0.0515
I-1-1	Basic nursing theory	0.3325	0.0727	
I-1-2	Clinical professional nursing knowledge	0.5278	0.1154	
I-1-3	Preventive health care and public health knowledge	0.1396	0.0305	
I-2	Humanities and social sciences knowledge	0.3333	0.1026	0.0515
I-2-1	Literature and art knowledge	0.2493	0.0273	
I-2-2	History and philosophy knowledge	0.1571	0.0172	
I-2-3	Social science knowledge	0.5936	0.0649	
II	Skills level	0.2065		<0.001
II-1	Teaching skills	0.3000	0.0619	0.0196
II-1-1	Carry out teaching design according to the objectives of curriculum humanities and students’ acceptance	0.3444	0.0227	
II-1-2	Combine specialty with humanities and guide students to think about humanities	0.2437	0.0161	
II-1-3	Is able to skillfully use modern teaching methods (video, PPT, etc.) to arouse students’ interest in curriculum humanities	0.1577	0.0104	
II-1-4	Ability to reflect and summarize the class	0.1577	0.0104	
II-1-5	Scientific research and innovation capability	0.0965	0.0064	
II-2	Humanistic skills	0.3000	0.0619	0.0227
II-2-1	Teamwork ability	0.3300	0.0218	
II-2-2	Information acquisition and application capability	0.3300	0.0218	
II-2-3	Observation and analysis ability	0.1996	0.0132	
II-2-4	Communication ability	0.1404	0.0093	
II-3	Clinical skills	0.2000	0.0206	0.0161
II-3-1	Ability to effectively evaluate the basic situation of patients	0.2952	0.0065	
II-3-2	Nursing diagnosis ability for diseases	0.1835	0.0040	
II-3-3	Nursing planning ability	0.2952	0.0065	
II-3-4	Ability to implement nursing operation	0.0878	0.0019	
II-3-5	Reasonably evaluate the nursing process, effect and realization of goals	0.1382	0.0030	
III	Professional attitudes	0.3077		<0.001
III-1	Professional cognition	0.3333	0.1026	<0.001
III-1-1	Professional values	0.6667	0.0729	
III-1-2	Professional thinking	0.3333	0.0365	
III-2	Professional emotion	0.6667	0.2052	0.0227
III-2-1	Native land emotion	0.1404	0.0307	
III-2-2	Sense of responsibility	0.3952	0.0864	
III-2-3	Caring for students	0.2322	0.0508	
III-2-4	Reverence for life	0.2322	0.0508	
IV	Personality traits	0.0890		<0.001
IV-1	Personal characteristics	0.6667	0.0594	0.0445
IV-1-1	Purposeful and determined	0.1095	0.0069	
IV-1-2	Calm and peaceful	0.2344	0.0148	
IV-1-3	Rigorous and serious	0.3654	0.0231	
IV-1-4	Set a good example for others	0.1502	0.0095	
IV-1-5	Self-confidence	0.1413	0.0089	
IV-2	Interpersonal traits	0.3333	0.0297	<0.001
IV-2-1	Approachability	0.1429	0.0045	
IV-2-2	Tolerance	0.2857	0.0090	
IV-2-3	Is good at listening	0.2857	0.0090	
IV-2-4	Empathy	0.1429	0.0045	
IV-2-5	Treat others equally and respect others	0.1429	0.0045	
V	Intrinsic motivations	0.0890		<0.001
V-1	Achievement motivations	0.3333	0.0297	<0.001
V-1-1	Sense of competence	0.2500	0.0079	
V-1-2	Interested in curriculum humanistic teaching	0.5000	0.0158	
V-1-3	Desire for self-improvement	0.2500	0.0079	
V-2	Affiliation motivations	0.6667	0.0594	<0.001
V-2-1	Others’ respect and recognition	0.6667	0.0422	
V-2-2	Sense of belonging	0.3333	0.0211	

## Discussion

Undergraduate nursing students are the backbone of future nursing development, and their humanistic qualities have an important impact on the quality of nursing services [5, 6]. Therefore, undergraduate nursing teachers need to improve their humanistic competencies to better cultivate undergraduate nursing students with humanistic care. In this study, we used the Delphi method to construct an evaluation index system for the humanistic competence of undergraduate nursing teachers. The evaluation index system was scientific and reliable. First, the experts included had rich experience in nursing education, clinical nursing management and medical humanities. In addition, the response rates in two rounds of consultation were above 88.89%, indicating that the experts had high enthusiasm [25, 29]. The Cr scores of the two rounds were more than 0.90, and Kendall’s W of the whole process was statistically significant (*P* < 0.05), which showed that the experts had good authority and that the consultation results were scientific and reliable [27].

Based on the onion model, using a systematic literature review and semi-structured interviews, this study constructed an indicator system from the 5 dimensions of knowledge base, skills level, professional attitudes, personality traits and intrinsic motivations [15]. Then, combined with Delphi consultation, we finally formed 5 first-level indicators, 11 s3cond-level indicators and 41 third-level indicators.

The first field “knowledge base” and the third field “professional attitude” had the highest weight in the two rounds of consultation; both are 0.3077, indicating that these two areas are the most important in the view of experts. In this study, experts considered that knowledge is the basis of “curriculum humanities” teaching, so undergraduate teachers should especially master professional nursing knowledge. This result is similar to previous studies [28, 30]. Furthermore, professional nursing knowledge is necessary, the weight is 0.6667 in the field of “knowledge base”. This result indicates that having a solid professional foundation is the fundamental for undergraduate nursing teachers to carry out curriculum humanistic teaching. Meanwhile, a foundation in the liberal arts is essential to prepare the kind of nurse who focuses on the complexity of the human experience [31, 32]. In this way, undergraduate nursing educators need to know and integrate relevant humanistic knowledge into the curriculum, including knowledge of literature and art, of history and philosophy, and of social science [4].

The “skills level” ranked second in the first-level indicators with a weight of 0.2065. It comprised “teaching skills”, “humanistic skills”, and “clinical skills”. In this dimension, the weight score of “teaching skills” and “humanistic skills” is 0.0619, and the weight of “clinical skills” ranks last, which is inconsistent with other studies [9, 27]. “Curriculum humanities” emphasizes that curriculum is the foundation, humanities is the focus, and teachers are the key [7]. Therefore, the landing point of curriculum humanistic competence should be inclined to teaching and humanistic skills. Compared to clinical skills, undergraduate nursing teachers should consciously improve their teaching and humanistic skills to ensure the quality of curriculum humanistic teaching. Undergraduate nursing teachers are supposed to have good teaching skills, such as teaching design, teaching guidance, teaching operation, teaching evaluation and scientific research innovation [28, 33], and be able to naturally integrate curriculum humanities into the teaching process. Meanwhile, because humanistic practice is the foundation of an essential attribute of nursing [34], undergraduate nursing teachers should master some practical humanistic abilities to be better qualified for curriculum humanistic teaching [35, 36]. For example, engaging in teamwork actively, interacting well with others and accumulating materials related to curriculum humanistic teaching in ordinary times and naturally integrating humanistic elements based on specific professional knowledge points. As for clinical skills, the requirements of curriculum humanities and expert consultation are not emphasized. However, nursing teachers have dual identities as teachers and nursing staff. Therefore, we selected five nursing procedures as the items of clinical practice ability.

Having a positive professional attitude, which was ranked first, similar to the knowledge base domain in the 2 rounds of Delphi consultation, is considered one of the most essential curriculum humanistic competences in China. The reason is that having a positive professional attitude is a basic element for medical workers in Chinese medical education opinions [27]. The scores of the secondary indicators “professional emotion” and “professional cognition” in this field are 0.2052 and 0.1026, ranking first and second among all secondary indicators. Having a sense of national pride and reverence for life is considered to be the unique professional emotion of Chinese undergraduate nursing teachers. This is because Chinese nursing workers have a lofty mission, and they should be ready to stand up when the motherland and people need them [5]. Additionally, undergraduate nursing educators need to care for students and be responsible for them, which is consistent with the research of other scholars [22, 37]. In terms of professional cognition, Undergraduate nursing teachers should possess good professional values and thinking, so that they can transmit these qualities to their students effectively, which will better prepare future nurses for the complex work situations they will encounter [12].

Good personality traits play an important role in the effect of curriculum humanistic teaching. Undergraduate nursing teachers should be confident and lead by example [22]. In this field, conscientious and emotional stability have the highest weights, with 0.3654 and 0.2344. As medical workers, Chinese experts believe that they need to have a firm will, maintain emotional stability, and treat nursing curriculum humanistic teaching with a rigorous and serious attitude [27]. For interpersonal traits, tolerance and good at listening ranked first, both were 0.2857. This may be related to the Confucian ideology in China, which focuses on “kindheartedness” and emphasizes harmonious teacher-student relationships. In this way, teachers should be tolerant, tireless in teaching, listen to others and help them. What’s more, students likely gravitate to instructors who are approachable, fair, open, and empathetic [9, 22]. Therefore, undergraduate nursing teachers should have these interpersonal characteristics so that students can better accept the ideas conveyed by curriculum humanistic teaching and ensure the quality of the course.

Intrinsic motivation is the core part of the competency model and plays a vital role in the evaluation of competence [14]. However, the weight scores of intrinsic motivation and personality traits are 0.0890, ranking last. The reason may be related to the fact that these dimensions belong to the implicit part of the onion model, which is not convenient to measure and evaluate [14]. In the dimension of intrinsic motivation, experts believe that affinity motivation has a strong impact on the humanistic curriculum competence of undergraduate nursing teachers, with a weight of 0.6667 in this field, including gaining respect from others and a sense of belonging [38]. Therefore, creating a harmonious atmosphere has a good promoting effect on curriculum humanistic teaching. Achievement motivations are an indispensable part of curriculum humanistic competence. It is necessary for undergraduate nursing teachers to have a passion for curriculum humanistic teaching and pursue lifelong learning to maintain curriculum humanistic competence [22, 23].

## Limitations

We had taken corresponding measures to maximize the response rate, but after two Delphi rounds, there was still a 20% nonresponse rate, which may affect the accuracy of the consultation. Although the experts came from 10 different provinces and municipalities, regional differences may exist. We tried to reduce such differences by selecting experts from China’s three major economic regions [39]. In the early stage, we mainly focused on qualitative research, and in the future, we will use quantitative research to improve the indicator system.

## Conclusions

A framework of curriculum humanistic competence, including the 5 dimensions of the knowledge base, skill level, professional attitude, personality traits and intrinsic motivations, was developed using a Delphi technique. This indicator system can provide targeted guidance based on weight of indicators for undergraduate nursing teachers to carry out humanities curriculum teaching and further improve the admission, assessment and evaluation system of undergraduate nursing teachers. Moreover, it can also provide new theories and strategies for other countries to build their own evaluation index system for curriculum humanistic competence to ensure the quality of undergraduate nursing education.

## Data Availability

The dataset in this study is available from the corresponding author on reasonable request.
